# Physical and social warmth

**DOI:** 10.1098/rsos.231575

**Published:** 2024-05-01

**Authors:** Haavard Koppang, Thorvald Harem, Lewend Mayiwar, Jaime Pineda

**Affiliations:** ^1^ Department of Leadership and Organizational Behaviour, BI Norwegian Business School, Oslo, 6281, Norway; ^2^ Department of Cognitive Science, University of California San Diego, , CA, 92037, USA

**Keywords:** awareness, self-regulation, embodiment, personality, IPIP-IPC

## Abstract

The concept of a warm person has played a key role in western social psychological research, particularly in how people perceive others. Williams and Bargh (2008; Study 1) found that individuals holding a cup of warm beverage perceived the individuals they faced as psychologically warmer than those who held a cup of cold beverage. In this article, we set out to replicate and extend these findings by exploring whether various factors modify the effect of physical and social warmth. Specifically, we tested three moderating variables: participants’ awareness of the purpose of the experiment, warmth of participants’ personality and the target person’s gender. We found no main effect of physical warmth, and very little evidence for any moderating effects. It is clear from this and other recent studies that the embodiment effect is not simple to replicate and, therefore, is difficult to exploit for practical purposes.

## 1. Introduction

The motivation for our work is to replicate and extend Williams and Bargh’s [1] study by exploring boundary conditions for the originally identified relation between physical and personal warmth. This seminal study by Williams and Bargh found that the experience of physical warmth increases feelings of interpersonal warmth without a person’s awareness of this influence. In one of their studies, participants who briefly held a cup of hot (versus iced) coffee judged a target person as having a ‘warmer’ personality (i.e. generous and caring). Consistent with embodiment theories, Williams and Bargh theorized participants embodied physical warmth and experienced it as social warmth. These findings have triggered speculations about similar effects of physical warmth on the perception of social proximity, interpersonal similarity and intimacy (e.g. [2,3]).

Citing both Harlow monkey studies and recent studies in neuroscience (e.g. [4–6]) Bargh [7] proposed an explanation for these results. He argued that the brain is hardwired to connect feelings of physical and social warmth; [5] added specificity to this explanation and claimed that social warmth and physical warmth share the same neural mechanisms. Using an fMRI scanner, they found that the participants’ insular cortex lights up both while holding a warm cup and while texting family members. Their discussion of the current state of research within the field led them to theorize that physical warmth is processed interoceptively and similar to the processing of affective states as social warmth (see also [4]). Bargh [7] concludes that these findings of the hardwiring of the connection between social and physical warmth and the individuals’ confounding of physical and social warmth show that evaluations of social warmth occur unconsciously; before you know it, so to speak, and bias our judgment of others.

This intriguing proposition leads us to explore boundary conditions for this stream of research: first, if the brain is hardwired to connect social and physical temperatures, will *awareness* of this connection modulate the evaluations of social warmth? And related to this, will individual differences, such as being a cold or warm person, or the target’s gender, modulate the connection between physical and social warmth?

The answers to these questions have both practical and theoretical implications. For example, if the hardwired effect can be overturned by making participants aware of the unconscious influence of physical warmth, there would be an easy way to overcome the bias. Theoretically, it would change the understanding of the information processing interacting with the insular cortex and the proposed hardwiring.

There have been attempts to replicate Study 2—holding a hot versus a cold therapeutic pad—from Williams and Bargh [1]. Lynott *et al*. [8] reported three attempts, although none of them produced the expected results that physical warmth increased the perception of interpersonal warmth. Lynott’s results were also in the opposite direction from those predicted by Williams and Bargh [1]. Wortman, Donnellan and Lucas [9] also reported difficulties in replicating hot/cold priming effects. These replication attempts neither controlled for awareness of the findings from the original studies, nor did they control for individual differences, as suggested in the present research. More recently, Chabris *et al*. [10] attempted a replication of the original work but failed to do so. Their study used more than triple the sample sizes (128 and 177) and double-blind procedures but found near-zero effects (*r* = −0.03 and 0.02). In both cases, Bayesian analyses suggested there was more evidence for the null hypothesis of no effect than for the original physical warmth priming hypothesis.

The Williams and Bargh study is well known among university students (e.g. among neuroscience and psychology students) and has increased participants’ awareness of the connection between physical warmth and perception of social warmth. Hence, in this article, we controlled for whether the awareness of the original Williams’ and Bargh’s finding moderates this effect of physical warmth on social warmth.

Incorporating awareness as a factor in our study aligns with recent discussions in replication studies and meta-science. In a published replication of the same study, Lynott *et al*. [8], the authors did not find support for the original effect. The authors argued that failing to replicate might be related to participants’ awareness. Expectancy effects have been extensively discussed in replication studies (e.g. [11]). Some suggest that effects observed in some original studies, including those by Williams and Bargh, may have been influenced by unconscious cues inadvertently conveyed by the researchers.

### 1.1. Theoretical foundations

One prominent and essential feature of our environment is temperature. Yet, only recently has research examined how temperature regulation and the experience of physical warmth affect social information processing [12]. In human social behaviour, one of the first impressions that leads to a variety of social attachments is whether someone is a ‘warm’ or ‘cold’ person. Indeed, some suggest that coldness and warmth are primary dimensions of social behaviour and are important for understanding interpersonal relationships [13,14]. These psychological dimensions appear to underlie group stereotypes in different countries and cultures and have a crucial role in how humans interact and work together.

Several relatively recent reports have supported the original observations of [1]. A recent study by Inagaki and Human [15] compared tympanic temperature, a measure of internal body temperature, with feelings of social connection assessed multiple times a day over one week. Consistent with the hypothesis that physical warmth and social warmth, or feeling socially connected to others are linked, changes in tympanic temperature covaried with feelings of social connection across assessments. A similar effect was reported outside of the lab using ‘daily diary’ methods. [16] found that for 235 participants going about their daily lives, on days when the participants reported feeling physically warmer (independently of the actual outdoor temperature), they also rated themselves as more interpersonally warm and agreeable.

In the Williams and Bargh study, participants were told a plausible cover story for holding cold or hot beverages. Firestone *et al*. [17] underline that the social nature of psychology experiments implies they are contaminated easily by task demands and that the subjects consciously or unconsciously adjust responses relative to experimenters’ desires. These investigators hold it is easy to counteract this type of effect by informing the participant directly about the experiment [17]. Therefore, awareness of the role of the hot and cold beverage might counteract the unconscious priming effect [18]. This is a question of whether subliminal conditioning effects are contingent on awareness, dating back to the mid-1950s [19].

In research on temperature regulation and the experience of physical warmth within the neurosciences, individual differences are not yet a topic of primary concern. However, within the field of social psychology, there is a great interest in research on individual differences, such as warm and cold personalities (e.g. [13,20,21]). Because securely attached personalities may correlate with dimensions of warm personality [20], it suggests a rationale for measuring warmth as a personality dimension. Theories of embodied cognition provide a practical explanatory framework for understanding the relationship between low-level physical sensations and higher-order psychological processes. In the dynamic interaction between perception, action and cognition, it is reasonable to assume that variations in personality play a moderating role. Individual personality traits reflect an array of distinct cognitive and social competencies that may impact the mapping between physical and psychological dimensions.

Bargh and Shalev [22] reported that higher scores on a measure of chronic loneliness (social coldness) were associated with an increased tendency to take warm baths or showers. Reciprocally, that a physical coldness manipulation significantly increased feelings of loneliness. Still, these individuals were more likely to choose a healthy snack and less likely to cheat, presumably to self-regulate the guilty feelings. Similarly, individuals who were induced to feel lonely tried to regulate these feelings of exclusion with a greater desire for warm drinks and food [23].

Personality researchers have argued that to sense a person as warmer or colder would require empathy—a trait associated with warmth—to recognize cues of social warmth [24]. This implies that a warmer person would be more sensitive to cues signalling social warmth. This rationale for including personality in our studies flows from observations such that securely attached children share more in a warm room than in a cold room [7,25]. In [26], where infants were raised alone with a wire or a soft terry-cloth maternal stand-in, baby monkeys preferred the soft warm experience over the wire mother. ‘If physical warmth can substitute for the missing social warmth in a person’s life, at least somewhat, then perhaps applications of physical warmth could be used as a cheap but effective therapy for emotional disorders, such as depression, which are often characterized by feelings of social isolation and decreased social connection (that is, social coldness)’ [7].

Others have explored this relation between the embodiment of physical warmth and personality in self-regulation [25,27,28]. The idea proposes that the tendency to maintain a balance regarding physical temperatures results in a similar homeostatic drive in the metaphorically embodied ‘interpersonal’ dimension. The outcome is a process of self-regulation that uses either physical (cf. bottom-up) or psychological (cf. top-down) features to regulate deviations from a state of balance. Suppose, we take personality to be the homeostatic stable state of multi-dimensional physical and psychological sensitivities. In that case, it is consistent to think that differences in these dynamics would affect the self-regulation of warmth or cold.

We cannot preclude that personality has an influence on the outcome of our studies, thus we have included measures of personality (cf. International Personality Item Pool–Interpersonal Circumplex or IPIP-IPC). Furthermore, our findings on participants’ personality might explain data that do not meet expectations, whereas a combination of personalities and beverages react differently to the target person than expected. Affect as embodied physical warmth experienced as social warmth of the target person might be about what is in mind and the object of judgment [29].

## 2. Methods

This study uses Williams’ and Bargh’s [1] study procedures—experiencing physical warmth promotes interpersonal warmth—and manipulates awareness of the effect of physical warmth on social warmth.

### 2.1. Sample

Williams’ and Bargh’s [1] Study 1 had a sample size of 41. We used student samples in our studies like Williams and Bargh. Our final sample comprised 127 participants. We conducted a sensitivity analysis in G*Power version 3.1.9.6 [30] to estimate the smallest effect that the current study could detect using the same analytical method as the original study by Williams and Bargh [1]. We excluded the participants who were in the condition that made them aware of the hypothesis and manipulation (i.e. the extension part of this replication). This resulted in a total sample of 65 participants for the sensitivity analysis. The sensitivity analysis in G*Power (test: ‘ANOVA: fixed effects, special, main effects and interactions’; α = 0.05, number of groups = 2, numerator d.f. = 1) showed that a sample of 65 participants gives the current study 80% power to detect a medium effect of η^2^ = 0.111. The current study should therefore be sufficiently powered to detect a medium effect size, as in the original study by Williams and Bargh. We computed the effect size in Williams and Bargh’s Study 1 based on the sample size (total *n* = 41) and F-statistics (F(1,39) = 4.08, *p* = 0.05), using the effectsize package in R [31]. This resulted in an effect size of Cohen’s *d* = 0.65 (which corresponds to *η* ^2^ = 0.09), which corresponds to a medium effect size. However, it is important to note that while our study should be sufficiently powered to detect a medium effect size, as seen in the original study by Williams and Bargh, original effects often tend to be inflated.

We collected the data in two locations. In Southern California, the data were collected towards the end and the early part of the year, whereas they were collected before and after the summer in Norway, and thus virtually under the same outdoor temperatures. Sixty-two participants (41 females) were recruited for the study in Southern California (age 19–29 years, mean 21.4, s. d. 1.7) from undergraduate programs in neuroscience and psychology. Sixty-five undergraduate students (31 females) were recruited as participants for the study in Norway—Northern Europe, from an undergraduate program in business and economics (age 19–38 years mean 22.1, s. d. 3.8).

All participants in California were offered course credit for their participation. The Internal Review Board (IRB) at the University of California, San Diego (UCSD) approved the protocol and gave informed consent to all participants under the Helsinki Declaration. For the study in Norway, participants were recruited without the offer of credit for their participation. They gave informed consent under the Helsinki Declaration, approved by a business school in Norway. Most of the participants in Norway were non-native English speakers, while most participants in California were native English speakers.

### 2.2. Materials, measures and manipulations

Participants were pseudo-randomly assigned to the Aware or Unaware condition (i.e. they were randomly assigned, but the assignment was constrained by the need to have approximately equal numbers in each condition). Sixty-two participants were assigned to the Aware condition, and 65 to the Unaware condition. Roughly 50% were sampled from each of the two study locations. In the Unaware condition, participants read an abstract that was irrelevant to the experiment and therefore remained unaware of the purpose of the study. In the Aware condition, participants read the Abstract from the original Williams and Bargh [1] paper. Doing so made them aware of the goal of the study.

Participants in California were greeted in the kitchen of the Cognitive Science Department at UCSD, which is on the ground floor, and asked to fill out a consent form and the IPIP-IPC scale (to measure individual differences in terms of warmth or coldness). The scores on the four aspects of the scale that reflect a WARM personality (aloof-introverted; unassured-submissive; unassuming-ingenuous; warm-agreeable) were averaged, as were the four that reflect a COLD personality (assured-dominant; arrogant-calculating; cold-hearted; gregarious-extraverted) [21]. Participants were then led to the main lab on the second floor. On the way to the lab, which took approximately 3–4 min via an elevator, they were asked to hold either a cup of hot tea (about 50°C/122°F) or ice-cold water (about 10°C/50°F). Subjects had to use a whole hand grip since cups did not have a handle to help while the experimenter asked and filled out a series of questions.

Participants in Norway were also pseudo-randomly assigned to an Aware or Unaware condition. We used the same procedures as used at UCSD at a business school in Norway. Participants were greeted in the kitchenette of the department of Leadership and Organizational Behavior and asked to fill out a consent form and the IPIP–IPC scale. On the way to the testing room, which took approximately 4 min via an elevator, participants were asked to hold a cup of hot tea (about 50°C/122°F) or cold water with ice cubes (about 10°C/50°F). During this time, the experimenter asked and filled out several questions. Once in the testing room, participants continued through the same procedures as the participants at a university in Southern California.

While in the lab/testing room, participants in the Aware condition read the abstract from the Williams and Bargh study [1], while those in the Unaware condition read an abstract about consumerism. We look at this as a manipulation of task demands, with the difference between conditions being aware of the study goals in one condition and unawareness in the other. All participants were then asked to fill out a Personality Impression Scale about an imaginary individual (to avoid gender biases, some subjects read a description of a male target person while others read about a female target person). We presented the imaginary individual using the same description as Williams and Bargh [1].

Descriptions of the individuals were provided in written form at the top of the scale (e.g. Person A is intelligent, skillful and industrious). (S)he is also determined, practical and cautious. Questionnaires asked participants to rate Person A on ‘the following 10 personality traits’ using a 7-point, bipolar Likert scale from positive (1 for happy) to negative (7 for unhappy). Following Asch and later Williams and Bargh, half of the personality traits (the first five questions) were semantically related to the cold-warm dimension, and the second half was not. In a debriefing questionnaire, participants were asked a series of questions about the purpose of the experiment, including the role of the hot tea/ice water. The goal was to assess their level of awareness of the original Williams and Bargh findings. Specifically, we asked participants the following open-ended questions: ‘What did the abstract you read mean to you?”, ‘What do you think was going on during the experiment?’ and ‘What was the role of the tea?’. Participants’ responses were then manually coded using a 5-point scale to indicate their level of awareness, ranging from 1 (unaware) to 5 (aware).

### 2.3. Analytical approach

The experiment was performed in two different locations: Southern California and Oslo. Because these locations are very different, for each key test, we tested moderation by location. It is important for readers to interpret these results with caution, however, as the current study may be underpowered to reliably detect interaction effects.

All analyses were performed in jamovi, version 2.4.8 [32]. We performed ANOVAs for the interaction between cup condition and awareness condition and for the interaction between cup condition and target gender. For the interaction between cup condition and warm and cold personality, we conducted a linear regression model. For the two latter moderation tests (moderation by personality and target gender), we only included participants in the Unaware condition.

To quantify evidence for the absence of an effect, we used Bayesian analysis and equivalence testing [33–35], we used the original effect size (Cohen’s *d* = 0.65) as the scaling factor for the Bayesian ANOVA tests. For the Bayesian linear regression models, we applied a beta (*a* = *b* = 1) prior to the models, which assumes that the model sizes are equally likely before observing any data, and we applied a JZS prior with an r scale of 0.325 (which corresponds to the scaling factor used in the Bayesian ANOVA [36]).

We report BF_01_, with values >1 indicating evidence for the null hypothesis over the alternative hypothesis: 1–3 (anecdotal evidence), 3–10 (moderate evidence), 10–30 (strong evidence), 30–100 (very strong evidence) and >100 (extreme evidence) [37,38].

## 3. Results

### 3.1. Awareness of the manipulation

An ANOVA indicated a main effect of awareness condition (*F*(1,123) = 175.82, *p* < 0.001, η_p_^2^ = 0.588, BF_10_ = 7.28 × 10^18^, BF_01_ = 0.00) and an interaction between awareness condition and location (*F*(1,123) = 4.64, *p* = 0.033, η_p_^2^ = 0.036, BF_10_ 6.90 × 10^23^, BF_01_ = 0.00). In the Southern California sample, the Unaware group scored on average 1.79 (s.d. = 1.02), while those in the Aware condition scored 4.43 (s.d. = .73). In the Norwegian sample, the Unaware group scored on average 1.24 (s.d. = 0.97), while those in the Aware condition scored 3.14 (s.d. = 1.09).

### 3.2. Effect of beverage temperature, awareness and location on perceived warmth

We ran an ANOVA that included cup condition, awareness condition, location and their interactions as predictors. The results are provided in table 1. There was evidence for no main effect of cup condition (Bayes factor indicated anecdotal evidence for the null hypothesis), no cup × awareness condition interaction (Bayes factor indicated strong evidence for the null) and no cup × awareness condition × location interaction (Bayes factor indicated extreme evidence for the null). There was indeterminate evidence for an interaction between cup condition and location. In the Southern California sample, the cold cup group scored 3.73 (s.d. = 0.82) and the hot cup group scored 3.28 (s.d. = 1.04), whereas in the Norwegian sample, the cold cup group scored 3.39 (s.d. = 0.78) and the hot cup group scored 3.53 (s.d. = 0.62).

**Table 1.** ANOVA model testing the effect of cup condition, awareness condition, location and their interactions.

predictor	*F*	*p*	η²p	BF_01_
cup	1.23	0.270	0.010	3.94
awareness	1.46	0.229	0.012	3.39
location	0.08	0.784	0.001	6.55
cup*awareness	1.93	0.168	0.016	26.66
cup*location	4.41	0.038	0.036	20.39
awareness*location	0.08	0.773	0.001	105.00
cup*awareness*location	0.93	0.336	0.008	1535.59

*Notes:* cup = cold cup (0), warm cup (1). Awareness = unaware (0), aware (1). Location = San Diego (0), Oslo (1).

### 3.3. Interaction between beverage temperature and rater’s personality

We ran a linear regression model that included cup condition, rater’s personality (warmth and cold), location and their interactions. The results are summarized in table 2. There was evidence for no main associations, no interactions with participants’ warm personality trait (Bayes factor indicated very strong evidence for the null) nor dominant/cold personality trait (Bayes factor indicated strong evidence for the null), no cup condition × warm personality × location interaction (Bayes factor indicated strong evidence for the null) and no cup condition × dominant/cold personality × location interaction (Bayes factor indicated extreme evidence for the null).

**Table 2.** Summary of multiple linear regression model including cup condition, rater’s personality (warm and cold personality traits) and their interactions as predictors.

predictor	*B*	SE	95% CI	*p*	BF_01_
intercept	3.68	0.37	3.20, 4.31	<0.001	
cup	−1.00	0.70	−2.24, 0.41	0.161	3.62
location	−0.18	0.44	−1.05, 0.69	0.681	3.49
rater warmth	0.14	0.32	−0.49, 0.77	0.659	3.48
rater cold	0.36	0.43	−0.51, 1.23	0.409	3.67
cup*rater warmth	0.77	0.66	−0.56, 2.10	0.251	17.83
cup*rater cold	−0.28	0.52	−0.31, 0.76	0.593	15.43
cup*location	1.17	0.77	−0.37, 2.71	0.133	15.02
rater warmth*location	−0.76	0.44	−1.65, 0.13	0.092	2.58
rater cold*location	−0.22	0.49	−1.21, 0.78	0.664	18.30
cup*rater warmth*location	−0.32	0.82	−1.96, 1.31	0.692	37.58
cup*rater cold*location	−0.02	0.61	−1.25, 1.20	0.973	89.29

*Notes:* cup = cold cup (0), warm cup (1), Location = San Diego (0), Oslo (1).

### 3.4. Interaction between beverage temperature and target’s gender

We ran an ANOVA model that included cup condition, target gender, location and their interactions as predictors (table 3). There was a main effect of target gender, suggesting that perception of social warmth was greater when the target was male rather than female. Bayes factor indicated moderate evidence in favour of the null hypothesis (BF_10_ = 8.34). There was no interaction between cup condition and target gender (Bayes factor indicated anecdotal evidence for the alternative hypothesis). Finally, the interaction between cup condition, target gender and location was not significant (Bayes factor indicated very strong evidence for the null).

**Table 3.** ANOVA model testing the effect of cup condition, target gender, location and their interactions.

predictor	*F*	*p*	η²p	BF_01_
cup	0.03	0.867	0.000	4.86
target gender	7.98	0.007	0.123	0.13
location	0.01	0.936	0.000	4.67
cup*target gender	1.82	0.183	0.031	0.95
cup*location	0.24	0.623	0.004	66.64
target gender*location	0.16	0.694	0.003	2.10
cup*target gender*location	0.02	0.895	0.000	145.58

*Notes:* cup = cold cup (0), warm cup (1), Location = San Diego (0), Oslo (1), Target gender = female (0), male (1).

## 4. Discussion

We set out to replicate and extend Williams’ and Bargh’s Study 1 by exploring whether various factors modify the effect of physical and social warmth. Specifically, we tested three moderating variables: participants’ awareness of the purpose of the experiment, participants’ personality and the target person’s gender. We did not find any evidence for a main effect, and weak evidence for moderating effects for participants’ awareness, participants’ personality or target person’s gender.

The issue of whether cognition has top-down effects (i.e. whether awareness influences the perception of social warmth) on the embodiment of physical energies is a fundamental question that is unsettled [17,39]. We do not find support for the idea that this sensory perception is ‘cognitively penetrable’. If we can replicate the embodiment effects, awareness about it may interrupt and even reverse such an effect. However, we did not find evidence for this. While awareness manipulation was a primary focus in the current study, we also examined potential moderation by participants’ personality traits (warmth and dominance) and target gender. However, none of these variables moderated the effect of physical warmth on perceptions of warmth.

On a more general note, ‘Reproducibility is the cornerstone of social science’ [40]. Indeed, it is a necessary foundational step for all scientific studies. The meta-study in *Science* finding that only 36% of replications had statistically significant results [41] concluded that there is room to improve reproducibility. Failures to replicate central studies in psychology have created more focus on the importance of such studies—including direct replication [42,43].

From another perspective, Barrett [44], after 3 years of unsuccessful attempts at replicating an experiment, did not think of this as an example of the replication crisis (cf. [45] on replication crisis and replicability of findings in psychology). To her, this is about how normal science works and how to learn from mistakes without moving on if findings do not hold up the second or even the eighth time. Her story points to the importance of context for the outcome of an experiment. In terms of a specific experiment, it is easy to forget about the context and ask if the experiment works or not, rather than trying to figure out under what conditions does it work?

Theories of embodied cognition provide an explanatory framework for understanding relationships between physical and social warmth. However, the strength and the direction of these effects have been inconsistent and may depend on the methods of investigation or the nature of the phenomena under investigation [10,46]. Recent studies have shown a relationship between ambient temperature and human social behaviour, such that physical warmth can increase feelings of warmth [1,2,22].

Others have explored the connection between the idea of embodiment of physical energies and personality in self-regulation [25,27,28]. The idea proposes that the tendency to maintain a balance regarding physical temperatures results in a similar homeostatic drive in the embodied ‘interpersonal’ dimension. The outcome is a process of self-regulation that uses either physical or psychological features to regulate deviations from a state of balance.

An interesting question arises as to why sometimes physical regulation (bottom-up) and at other times psychological (top-down) regulation dominate the process. When does physical regulation dominate, and when does psychological regulation dominate? The dynamics of this interaction are undoubtedly complex and answers to these questions may be a factor that complicates replication studies. Future research should explore such factors.

There seems to be a belief that ‘the link between the experience of physical warmth and interpersonal warmth has been widely demonstrated’ [47]. Still, publications trying to replicate Williams and Bargh [1] failed to do so (cf. [8–10,48]). Williams’ and Bargh’s findings [1] have been cited 2033 times so far, and the citation trend over the years 2008–2022 is stable.

The present study investigated the proposed embodiment of physical temperature from holding a hot and cold cup and its effect on the perception of social warmth. We attempted to replicate Williams’ and Bargh’s [1] study, which had a sample of 41 participants in two conditions (hot and cold cup). We did not find a consistent pattern supporting the original findings, Bayesian analysis and equivalence testing also provided evidence for the absence of an effect. Given the relatively small sample in the current study, however, we interpret the results as inconclusive.

There could be other factors involved that we have not controlled for. Current studies of embodiment mechanism of physical warmth have not controlled for differences in environmental temperatures. That is, outside the building or inside the lab and whether participants feel warm or cold when they enter the lab. The reference-point such atmospheric conditions provide may influence whether and how holding a warm cup is associated with the embodiment mechanism. Perhaps if the participant is very warm and gets to hold a warm cup, it does not translate to making the target person any warmer. Maybe a very warm person will enjoy holding a cold cup and associate such positive characteristics with the target person. In summary, we think that the effects of the ‘hot’ and ‘cold’ cup for some parts are determined by a blending of participants, experimenters, the situation and atmospherics of the lab, and outside environment, since the effects investigated appear sensitive to such influences. Separating these effects is a challenge for future studies.

## 5. Conclusions

Our conclusion as to Williams’ and Bargh’s important study from 2008 is clear: to recreate factors that can reproduce the embodiment effect of physical warmth is not straightforward and unproblematic. Whether embodiment effects depend on task demands has not been addressed (for reviews, see [17]). Awareness of the study and its intention was for some participants triggered by a dominant intention not to be deceived by what they had noticed. However, we did not find evidence for a main effect, nor any moderating effect by awareness.

Embodiment effects are small in effect size, making them fragile and not likely to compete with explicit motives and goals. It is clear from this and other recent studies that the embodiment effect is difficult to replicate—and therefore, it is not easy to exploit for practical purposes. Given the unpredicted and random pattern of results, it is also likely that our results reflect random noise in the absence of any coherent effect. This would suggest that the embodiment effect itself is not real.

## Data Availability

Data and code are available on the Open Science Framework [49].
